# Supplementary figures and images for: Corticospinal excitability remains unchanged in the presence of residual force enhancement and does not contribute to increased torque production
Source: PeerJ. 2022 Jan 6;10:e12729. doi: 10.7717/peerj.12729 (PMC8743010; doi:10.7717/peerj.12729)

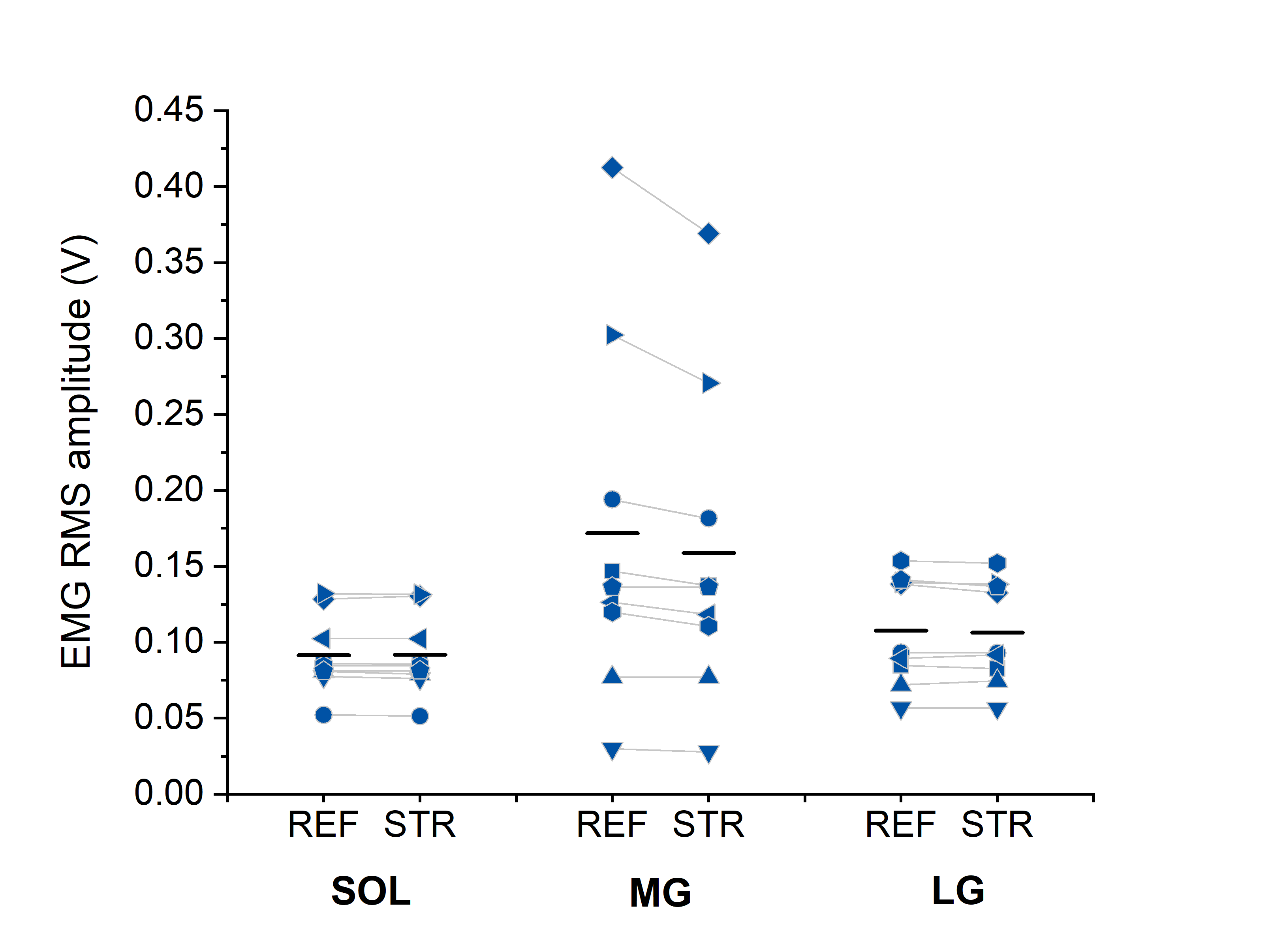

Supplement: Supplemental Information 1 — Background EMG amplitudes from the triceps surae muscles following subthreshold TMS for individual participants (blue symbols and grey lines) and the group average (black horizontal lines) during active stretch-hold (STR) and fixed-end reference (REF) contractions. [file peerj-10-12729-s001.png]
