# Supplementary material for: Corticospinal excitability remains unchanged in the presence of residual force enhancement and does not contribute to increased torque production
Source: PeerJ. 2022 Jan 6;10:e12729. doi: 10.7717/peerj.12729 (PMC8743010; doi:10.7717/peerj.12729)
Supplement: Supplemental Information 2 — Virtual data to demonstrate the effect of altering maximal M-wave (M_max) amplitudes (e.g., due to peripheral excitability changes or configurational changes of the EMG electrodes relative to the underlying muscle fibres) and/or MEP size and/or CMEP size on the MEP/CMEP ratio. In the first virtual test condition, MEPs and CMEPs are reduced by the same amount between reference and test conditions (from 10 to 5), which results in a MEP/CMEP ratio of 1 indicating reduced spinal excitability, but unchanged cortical excitability. However, as the M_max corresponding to the MEPs changed as well (the M-wave MEP became smaller by 20% between reference and test conditions, whereas the M-wave CMEP was unchanged), the normalised MEP/CMEP ratio goes up to 1.25. This virtual finding would subsequently indicate reduced spinal excitability, but increased cortical excitability. In the second virtual test condition, CMEPs are reduced between reference and test conditions (again from 10 to 5), but MEPs are unchanged. While the reduced CMEPs would again indicate reduced spinal excitability, the increased MEP/CMEP ratio of 2 (as expected based on reduced CMEPS, but unchanged MEPs) would indicate increased cortical excitability. However, this calculated ratio might be biased by M-waves varying between conditions. Accordingly, the two test conditions demonstrate that it is virtually impossible to interpret MEP/CMEP ratios when the corresponding M_max changes are unknown. [file peerj-10-12729-s002.docx]

|  | **absolute** | **normalised** | **absolute** | **normalised** | **absolute** | **normalised** |
| --- | --- | --- | --- | --- | --- | --- |
| **Response** | **Reference condition** | | **Test condition 1** | | **Test condition 2** | |
| **Mmax_MEP** | 10 |  | 8 |  | 8 |  |
| **MEP** | 10 | 1 | 5 | 0.625 | 10 | 1.25 |
| **Mmax_CMEP** | 10 |  | 10 |  | 10 |  |
| **CMEP** | 10 | 1 | 5 | 0.5 | 5 | 0.5 |
| **MEP/CMEP** | 1 |  | **1** |  | **2** |  |
| **MEPnorm/CMEPnorm** | 1 |  | **1.25** |  | **2.5** |  |
